# Post-traumatic Stress Disorder: A Narrative Review of Pharmacological and Psychotherapeutic Interventions

**DOI:** 10.7759/cureus.44905

**Published:** 2023-09-08

**Authors:** Mohammad Mansour, Geethi Rose Joseph, Golda K Joy, Shandesh Khanal, Rachana Reddy Dasireddy, Aardra Menon, Iyesatu Barrie Mason, Janvi Kataria, Tirath Patel, Shivani Modi

**Affiliations:** 1 General Medicine, University of Debrecen, Debrecen, HUN; 2 General Medicine, Jordan University Hospital, Amman, JOR; 3 General Medicine, Father Muller Medical College, Ontario, CAN; 4 General Practice, St. John's Medical College, Bengaluru, IND; 5 General Practice, Lumbini Medical College, Kathmandu, NPL; 6 General Practice, AMA School of Medicine, Makati, PHL; 7 General Practice, PK Das Institute of Medical Sciences, Kerala, IND; 8 General Medicine, Johns Hopkins Bloomberg School of Public Health, Baltimore, USA; 9 School of Medicine, D.Y. Patil University, Mumbai, IND; 10 School of Medicine, American University of Antigua, St. John's, ATG; 11 Internal Medicine, Einstein Healthcare Network, Philadelphia, USA

**Keywords:** evidence-based psychotherapy, prolonged exposure, benzodiazepam, selective serotonin reuptake inhibitor (ssri), cognitive behavioural therapy (cbt)

## Abstract

Post-traumatic stress disorder (PTSD) is a complex mental health condition affecting individuals exposed to traumatic events. This paper is a narrative review of the existing literature on pharmacological and psychotherapeutic interventions for PTSD. Treatment includes selective serotonin reuptake inhibitors (SSRIs), serotonin-norepinephrine reuptake inhibitors (SNRIs), and alpha-1 adrenergic receptor antagonists. By exploring the outcomes of these interventions, the review seeks to provide valuable insights into their potential as PTSD treatment options. The paper also highlights the importance of tailoring treatment plans to individual needs and discusses emerging treatments, such as mindfulness-based therapies, virtual reality therapy, and neurostimulation techniques. By integrating findings from various studies, it aims to offer valuable information to optimize treatment strategies and enhance outcomes for individuals suffering from PTSD. The goal is to support informed decision-making, ultimately leading to more effective and tailored approaches to address the challenges posed by this debilitating condition.

## Introduction and background

Post-traumatic stress disorder (PTSD) is a mental health condition that can develop after experiencing or witnessing a traumatic event. Common triggers for PTSD include combat exposure, physical or sexual assault, natural disasters, accidents, or other life-threatening situations. Symptoms of PTSD may include flashbacks, nightmares, intrusive thoughts, hyperarousal, avoidance of reminders of the trauma, and emotional numbness. It can significantly impact a person's daily life, relationships, and overall well-being. PTSD is a treatable condition, and effective interventions like trauma-focused psychotherapies (TFP) and pharmacological treatments are available to help individuals cope with and recover from the effects of trauma. Early recognition and appropriate support can make a substantial difference in the recovery process [1-5].

PTSD is a psychiatric condition characterized by significant impairment and limitations in daily functioning. It involves a cluster of symptoms, including intrusive recollections, avoidance behaviors, negative thoughts and emotions, and heightened arousal, persisting for an extended period after exposure to highly distressing events [1]. The prevalence of PTSD in the general population is estimated to be approximately 6-8%. However, this rate significantly increases in specific subpopulations exposed to severe psychological trauma. Military veterans, displaced individuals, and assault survivors, for instance, may experience much higher rates of PTSD, reaching as high as 25% [2].

Effective treatments for PTSD include pharmacological interventions and various psychotherapeutic approaches, but achieving evidence-based prescribing remains challenging due to inconsistent findings and recommendations from previous reviews [3]. TFP, such as prolonged exposure (PE), cognitive processing therapy (CPT), cognitive therapy for PTSD (CT-PTSD), and eye movement desensitization and reprocessing (EMDR), are widely recommended and supported by robust evidence as the preferred initial treatment for PTSD [4-6]. However, some individuals with PTSD may struggle to actively participate in these therapies due to heightened distress and emotional numbing within their optimal arousal zone [7]. This has led to considerable dropout rates and suboptimal responses in clinical trials [8,9].

Pharmacological treatments have shown a modest reduction in PTSD symptom severity [3]. However, given their limited success, there has been growing interest in investigating the combination of pharmacological agents with psychotherapies for PTSD [10]. The exploration of combined treatments holds promise in optimizing therapeutic outcomes for individuals with PTSD, but more research is needed to better understand their efficacy and safety in different populations.

This review paper addresses the problem of comprehensively evaluating the existing literature on pharmacological and psychotherapeutic interventions for PTSD. The primary objectives of this narrative review are to assess the effectiveness and safety of these interventions, identify potential gaps and limitations in current research, and propose recommendations for future studies. By achieving these goals, this review aims to contribute to a deeper understanding of PTSD treatment options and provide valuable insights and guidance for clinicians and researchers in their therapeutic practices.

## Review

We used a thorough strategy to compile pertinent material for this narrative review of PTSD therapies, conforming to accepted norms and principles for narrative reviews. Electronic databases like PubMed, PsycINFO, EMBASE, and the Cochrane Library were all thoroughly searched by our team. To find possibly neglected papers, we also manually searched reference lists from systematic reviews, meta-analyses, and important articles.

PTSD is a mental health condition that can develop after experiencing or witnessing a traumatic event. Common triggers for PTSD include combat exposure, physical or sexual assault, natural disasters, accidents, or other life-threatening situations. Symptoms of PTSD may include flashbacks, nightmares, intrusive thoughts, hyperarousal, avoidance of reminders of the trauma, and emotional numbness. It can significantly impact a person's daily life, relationships, and overall well-being. PTSD is a treatable condition, and effective interventions like TFP and pharmacological treatments are available to help individuals cope with and recover from the effects of trauma. Early recognition and appropriate support can make a substantial difference in the recovery process [1-4].

PTSD is a psychiatric condition characterized by significant impairment and limitations in daily functioning. It involves a cluster of symptoms, including intrusive recollections, avoidance behaviors, negative thoughts and emotions, and heightened arousal, persisting for an extended period after exposure to highly distressing events [5]. The prevalence of PTSD in the general population is estimated to be approximately 6-8%. However, this rate significantly increases in specific subpopulations exposed to severe psychological trauma. Military veterans, displaced individuals, and assault survivors, for instance, may experience much higher rates of PTSD, reaching as high as 25% [2].

Effective treatments for PTSD include pharmacological interventions and various psychotherapeutic approaches, but achieving evidence-based prescribing remains challenging due to inconsistent findings and recommendations from previous reviews [3]. TFP, such as PE, CPT, CT-PTSD, and EMDR, are widely recommended and supported by robust evidence as the preferred initial treatment for PTSD [4-6]. However, some individuals with PTSD may struggle to actively participate in these therapies due to heightened distress and emotional numbing within their optimal arousal zone [7]. This has led to considerable dropout rates and suboptimal responses in clinical trials [8,9].

Pharmacological treatments have shown a modest reduction in PTSD symptom severity [9]. However, given their limited success, there has been growing interest in investigating the combination of pharmacological agents with psychotherapies for PTSD [10]. The exploration of combined treatments holds promise in optimizing therapeutic outcomes for individuals with PTSD, but more research is needed to better understand their efficacy and safety in different populations.

This review paper addresses the problem of comprehensively evaluating the existing literature on pharmacological and psychotherapeutic interventions for PTSD. The primary objectives of this narrative review are to assess the effectiveness and safety of these interventions, identify potential gaps and limitations in current research, and propose recommendations for future studies. By achieving these goals, this review aims to contribute to a deeper understanding of PTSD treatment options and provide valuable insights and guidance for clinicians and researchers in their therapeutic practices.

Pharmacological interventions for PTSD

Pharmacological interventions have demonstrated promising results in mitigating the severity of PTSD symptoms, although their overall impact is relatively modest. A recent comprehensive review and meta-analysis of 21 trials conducted in 2021 supported the effectiveness of selective serotonin reuptake inhibitors (SSRIs) such as paroxetine, fluoxetine, and sertraline, along with the serotonin-norepinephrine reuptake inhibitor (SNRI) venlafaxine, and the atypical antipsychotic quetiapine as standalone treatments for PTSD. Following the PRISMA guidelines, this review identifies the FDA-approved treatment for PTSD as "sertraline," a selective SSRI. The efficacy of sertraline in treating PTSD is supported by a meta-analysis of randomized controlled trials, revealing a response rate of approximately 50-60% among individuals receiving sertraline therapy [11,12].

In a comprehensive systematic review and meta-analysis published in 2016, researchers aimed to identify the first-line treatment for PTSD by comparing psychotherapeutic and pharmacotherapeutic options. The study revealed that TFP consistently outperformed medications in the treatment of PTSD. While medications primarily alleviate PTSD symptoms, they do not seem to directly address the underlying neurobiological pathways crucial for extinguishing the patient's conditioned fear response, a key focus during cognitive-behavioral therapy (CBT). However, for individuals experiencing heightened autonomic activation during psychotherapy, sertraline (an SSRI) or venlafaxine (an SNRI) may be more suitable alternatives [14]. Overall, psychological interventions were found to be more effective than pharmacological interventions in treating PTSD [15]. Table 1 provides a brief overview of the different classes and mechanisms of action of drugs commonly used for PTSD treatment.

**Table 1:** Commonly used PTSD medications PTSD: post-traumatic stress disorder, MOA: mechanism of action, SSRI: selective serotonin reuptake inhibitor, SNRI: serotonin-norepinephrine reuptake inhibitor [12]

Class	MOA	Examples	Therapeutic aim
Anti-depressants	SSRI	Paroxetine	Symptoms improvement [5,12}
		Sertraline	
		Fluoxetine	
	SNRI	Venlafaxine	Improving overall PTSD symptoms [5}; potentially less effective in managing symptoms of heightened arousal [12}
	Serotonin-5-HT_2_ and adrenoreceptor α_2_ antagonism	Mirtazapine	Improving overall PTSD symptoms [5,13]
Anxiolytics/sedative hypnotics	GABA_A_-receptor antagonism	Alprazolam, clonazepam	No evidence of efficacy, with possible worsening of symptoms [14-17]
	α_1 _adrenoreceptor antagonism	Prazosin	Mainly used for sleep symptoms and may improve other symptoms [16]
Antipsychotics (second generation)	Dopamine D_2 _and serotonin-5-HT_2 _antagonism	Risperidone, quetiapine	

Emerging approaches but not FDA-approved

These medications fall into two main categories: conventional pharmacological agents, like SSRIs and SNRIs, and unconventional agents such as 3,4-methylenedioxymethamphetamine (MDMA) [11,12]. A systematic review and meta-analysis of MDMA utility in assisted psychotherapeutic intervention have shown that it results in greater reductions in clinician-administered PTSD scale (CAPS) scores compared to control psychotherapy. Furthermore, MDMA-assisted psychotherapy increased the likelihood of achieving clinically significant reductions in CAPS scores (relative risk 3.65; 95% CI, 2.39-5.57). It was also shown to result in score reductions that are sufficient to no longer meet the PTSD diagnostic criteria (relative risk, 2.10; 95% CI, 1.37-3.21) [12]. By modulating fear responses, MDMA can improve psychotherapy effectiveness and outcomes for PTSD by fostering a psychological state that promotes the exploration and processing of traumatic experiences more favorably [13].

Exploring different classes of medications for PTSD treatment

The most widely studied drug class for PTSD treatment is SSRI with the largest number of clinical trials available. Only two SSRI medications, paroxetine and sertraline, have received US FDA approval. SSRIs are considered to be the first-line prescribed medication [15], as they have been shown to elicit a response in approximately 60% of patients with PTSD [14]. Based on a comprehensive systematic review and meta-analysis of relevant literature, sertraline, an SSRI, demonstrated superior efficacy compared to other medications commonly used in PTSD treatment. However, the results for paroxetine and fluoxetine, also SSRIs, showed only marginal improvements. Surprisingly, citalopram use did not exhibit a significant difference when compared to a placebo. Desipramine, fluoxetine, paroxetine, phenelzine, risperidone, sertraline, and venlafaxine showed more positive effects compared to fake treatments (placebos) for treating PTSD. Among them, phenelzine stood out as better than many other active treatments. It was also the only drug that significantly worked better than the placebo in terms of fewer people stopping the treatment (dropouts). The numbers showed that the likelihood of dropping out was 7.50 times higher with a placebo than with phenelzine, with a range of certainty from 1.72 to 32.80. Mirtazapine had a relatively good score for effectiveness, but its score for being well-tolerated was not among the best. Divalproex had the lowest ranking overall, suggesting it was the least effective among all the treatments. These findings highlight the varying effectiveness of different SSRIs, emphasizing the necessity for future comparative studies to thoroughly analyze the efficacy of each relative to the others [16].

Similar to sertraline, venlafaxine showed better results than other medications. Therefore, it is often considered a first-line treatment alongside SSRIs, although evidence of its effect has not been consistently demonstrated in all clinical trials [15]. In addition, despite demonstrating a valuable initial clinical improvement, its effect appeared to diminish beyond 12 weeks. Benzodiazepines (BZDs), which act on gamma-aminobutyric acid (GABA)-A receptors, can potentially disrupt the process of memory consolidation, which could interfere with forming associations related to fear after a traumatic event [16].

Nonetheless, there is an ongoing debate regarding the effectiveness of BZDs as symptomatic treatments for anxiety, insomnia, and irritability in individuals with PTSD. A systematic review and meta-analysis study of 18 clinical trials and observational studies involving 5,236 participants published in 2014 aimed to provide a comprehensive analysis of the effectiveness of BZDs in PTSD treatment. The results revealed that BZDs are ineffective for PTSD treatment and prevention, with associated risks outweighing short-term benefits [15-17].

Based on a systematic review and meta-analysis of relevant studies published in 2020, treatment with prazosin, an antihypertensive and alpha-1 adrenergic antagonist, yielded modest improvements in PTSD symptoms. In this study, prazosin was the only pharmacological intervention with sufficient data to conduct a meta-analysis. Those findings suggested the need to reconsider recommendations against its usage [17].

The FDA has approved sertraline and paroxetine as the primary medications for PTSD. Typically, SSRIs are the first-line treatment for this condition, but exceptions may arise based on individual factors such as side effects, response to treatment, presence of other conditions, and personal preferences. For instance, a patient with PTSD and comorbid bipolar disorder might require caution with antidepressants due to potential mood instability. In such cases, it might be beneficial to administer a mood-stabilizing medication like lithium or an anti-epileptic drug before considering an SSRI [18-20].

The treatment of PTSD in children and adolescents requires careful consideration because they are still developing. We should discuss some safety concerns, such as potential therapeutic effects on brain growth. The effects of treatment throughout adolescence may endure a lifetime and affect re-traumatization risk, future mental health, and brain plasticity. The treatment of the elderly presents a unique set of challenges due to potential comorbidities and age-related sensitivities. It's crucial to consider any potential interactions between these medications and PTSD therapy. If cognitive decline or dementia is present, questions are raised about the patient's ability to manage their medications or fully participate in therapy. For each of these populations, rigorous risk-benefit evaluations are necessary [18-20].

Psychotherapeutic interventions: approaches and effectiveness

Numerous psychological treatments are available for PTSD. They are broadly grouped into two categories: trauma-focused and non-trauma-focused interventions. Trauma-focused treatments aim to confront and process memories, emotions, and thoughts related to the traumatic event. Examples include PE and CPT. On the other hand, non-trauma-focused treatments target the symptoms associated with PTSD without directly addressing the specific traumatic experience. These approaches encompass relaxation techniques, stress inoculation training, and interpersonal therapy. The main objective is to mitigate the impact of PTSD symptoms while avoiding direct engagement with the traumatic memories and emotions [21].

EMDR and CBT are two evidence-based psychotherapies proven effective in treating PTSD by addressing disturbing memories and maladaptive thoughts. EMDR consists of several phases, including history taking and treatment planning, preparation and coping strategies development, traumatic incident visualization and cognition assessment, desensitization and reprocessing using dual attention stimuli, creating positive cognitions, body scans for residual symptoms, and relaxation techniques, followed by a reevaluation phase. EMDR utilizes the orienting response and working memory hypothesis to modify responses to traumatic stimuli, resulting in reduced emotional impact. Additionally, it mimics the effects of REM sleep, facilitating the reorganization of traumatic memories mediated by the hippocampus and amygdala [22-25].

Conversely, CBT is typically delivered over four weeks, with sessions lasting up to 120 minutes. It primarily focuses on cognitive restructuring and exposure therapy. CBT helps patients recognize cognitive distortions and avoidance behaviors related to the trauma and guides them through imaginal exposure and relaxation exercises to reduce anxiety. Both therapies have demonstrated equal efficacy in treating PTSD when administered by skilled psychotherapists. Early recognition and prompt initiation of treatment have been associated with improved outcomes [26].

PE is another recommended treatment for PTSD based on the emotional processing theory. PE aims to modify pathological fear structures linked to traumatic memories by systematically confronting trauma reminders through in vivo and imaginal exposures [27-29].

CT-PTSD is rooted in Ehlers and Clark's cognitive model of PTSD, which focuses on negative appraisals of trauma and disturbances in memory. The treatment aims to modify negative appraisals, correct autobiographical memory, and change maladaptive cognitive and behavioral strategies used to cope with trauma-related distress [30]. Similarly, CPT is also recommended for PTSD treatment and is based on SCT. CPT identifies and challenges maladaptive assimilated and over-accommodated beliefs related to the trauma, shifting them toward adaptive accommodated beliefs [31,32].

A study comparing PE and CPT found that both treatments resulted in meaningful decreases in PTSD severity, with PE showing slightly higher effectiveness. For each treatment, the central psychological change mechanisms involve modifying maladaptive beliefs and perceptions associated with the traumatic event [33].

PE, CPT, and CBT are all recommended treatments for PTSD. Each therapy addresses traumatic memories or thoughts related to the trauma. Although research does not conclusively favor one over the others, they are considered first-line treatments for PTSD, taking into account patient preferences and clinician expertise [33].

An adjunctive treatment for PTSD is art therapy, a non-pharmacological complementary therapy, which has shown promising clinical effects on mental disorders. It involves using art media as a means of communication in psychotherapy. Art therapy provides a safe way to approach traumatic memories by using symbols to express emotions and reactions related to trauma. Traumatic experiences can be transformed into linguistic communication, allowing patients to externalize and distance themselves from the trauma. Studies have indicated that art therapy when combined with CPT can lead to improved trauma processing and reduced depression and PTSD symptoms in combat-related PTSD among veterans [33].

While various psychosocial treatments for PTSD have been effective for many, a significant number of patients still experience residual symptoms. Brief treatments that address faulty cognitions or maladaptive coping strategies may indirectly foster exposure and help patients confront their fears more openly. Guided mindfulness meditation has also been found beneficial for PTSD patients, assisting them in managing negative emotions that cognitive therapies may evoke [31].

Some of the established treatments are CBT, which includes cognitive restructuring and exposure therapy, which are well-established treatments for PTSD. It focuses on addressing maladaptive thoughts and avoidance behaviors related to the trauma. CBT has a robust evidence base and has demonstrated effectiveness in treating PTSD. EMDR is another evidence-based psychotherapy for PTSD. EMDR has gained substantial support for its efficacy in treating PTSD. PE has a solid evidence base and is considered an effective treatment for PTSD. CPT has been found effective in treating PTSD and has a notable evidence base. CT-PTSD emphasizes exposure and cognitive restructuring techniques. It's considered an evidence-based treatment for PTSD. Art therapy, while not as extensively researched as the aforementioned therapies, has shown promising clinical effects as an adjunctive treatment for PTSD [25-31].

The response rates for these treatments can vary depending on individual factors, such as the severity of PTSD, the duration of symptoms, and patient engagement. Generally, research suggests that a significant proportion of individuals experience meaningful reductions in PTSD symptoms with these evidence-based treatments.

There is evidence to suggest that combining psychotherapy (such as CBT, EMDR, PE, or CPT) with pharmacotherapy (medications) can lead to improved outcomes for some individuals with PTSD. Combining these approaches can potentially target different aspects of the disorder, such as cognitive and emotional processing. However, the decision to use combined therapy should be made on a case-by-case basis, taking into account the patient's preferences, the severity of symptoms, potential interactions, and individual response to treatment [28-31]. Monotherapy with evidence-based psychotherapies can also be effective for many individuals with PTSD. Research supports the notion that treatments like CBT and EMDR can lead to significant symptom reduction when administered by skilled professionals. It's essential to consider patient preferences and the expertise of the treating clinician when deciding on the most appropriate treatment approach.

In summary, evidence-based psychological treatments for PTSD, such as CBT, EMDR, PE, CPT, and trauma-focused CBT, have demonstrated efficacy in addressing PTSD symptoms. Response rates can vary, and the choice between combined therapy and monotherapy should be tailored to the individual's needs and circumstances. Art therapy, while promising, may have a less established evidence base compared to the others but can still offer benefits as an adjunctive treatment.

Challenges in implementing alternative therapies in real-world settings

The available primary studies on alternative pharmacotherapy strategies for PTSD have often been small and lacking sufficient descriptions, making replication challenging. Variability in dose, duration, and frequency of interventions further complicates the interpretation of findings, resulting in limited scientific evidence [11]. Additionally, due to the unfamiliar mechanisms of action, healthcare providers may express skepticism, impeding the integration of alternative pharmacotherapy into mainstream healthcare practices [29]. Patient acceptance and compliance may also pose challenges, as doubts about the efficacy of these approaches can lead to reduced treatment adherence. High rates of dropout in studies highlight potential problems with adherence to certain treatments [32].

Moreover, unlike conventional drugs, many alternative pharmacotherapy options lack stringent regulations and standardization, which raises concerns about product quality and consistency. Additionally, the potential for drug interactions between alternative pharmacotherapy and conventional medications may result in adverse effects or reduced efficacy, and patients might not always be fully aware of these potential risks. Another hurdle is that health insurance plans often do not cover alternative pharmacotherapy, making it financially burdensome for patients and limiting accessibility. Furthermore, healthcare providers may lack sufficient knowledge and training in alternative pharmacotherapy, affecting their ability to recommend and monitor these treatments effectively [33].

Certain regions or healthcare settings might not readily offer alternative pharmacotherapy, especially in underserved areas [28]. Similarly, cultural beliefs and societal attitudes toward non-conventional treatments can influence the acceptance and adoption of alternative pharmacotherapy [29]. Successfully integrating alternative pharmacotherapy into existing healthcare systems may require significant changes in protocols, guidelines, and collaboration among healthcare providers. The use of alternative pharmacotherapy also raises ethical questions concerning informed consent, transparency, and ensuring patients are well-informed about potential risks and benefits [30-33].

Future recommendations for PTSD treatment

The field of PTSD treatment faces several research gaps that require further investigation to improve interventions and outcomes. Firstly, optimizing exposure-based TFP like CBT and EMDR requires identifying the most effective treatment components, dosages, and sequencing, as well as understanding which subgroups of individuals benefit more from specific approaches. Longitudinal studies are essential to assess the long-term outcomes of PTSD treatment, including maintenance of treatment gains and factors contributing to sustained recovery. Research should also examine the impact of treatment on other domains, such as social, occupational, and relational functioning.

Exploring novel therapeutic approaches, such as mindfulness-based therapies, pharmacological treatments, virtual reality therapy, or neurostimulation techniques, through rigorous studies can add to the treatment options available for PTSD. In addition, personalized interventions should be explored, including identifying biomarkers, genetic factors, or other predictors to tailor treatments to individual needs and characteristics. Research on matching individuals to appropriate interventions based on these factors can lead to more effective and targeted treatments.

To bridge the research-practice gap and barriers, facilitators to the implementation of evidence-based interventions need investigation. Strategies should be developed to promote widespread adoption and sustainability of effective treatments in clinical practice.

Cultural factors and intersectionality should also be considered in treating PTSD. Culturally adapted interventions and understanding cultural influences on treatment outcomes can reduce disparities and improve treatment effectiveness for diverse populations.

Implementing pharmacological and psychotherapeutic interventions in real-world settings can face challenges. Limited access to mental health services, stigma, treatment adherence, and provider training are factors that need to be addressed. Comorbid conditions, such as depression and substance use disorders, complicate treatment approaches and require integrated care for better outcomes. Systemic barriers, like limited resources and coordination between primary care and mental health services, reimbursement limitations, and cultural factors, also impact implementation. Addressing these challenges requires a comprehensive approach involving system improvements, provider support, patient education, and policy changes.

By addressing these research gaps and challenges, the field of PTSD treatment can make significant strides in improving interventions and ensuring better outcomes for individuals affected by PTSD.

## Conclusions

In conclusion, this comprehensive review underscores the potential effectiveness of TFP and pharmacological approaches in alleviating PTSD symptoms and improving the quality of life for individuals with PTSD. The findings highlight the importance of conducting rigorous research to identify the most optimal treatment components, dosages, and sequencing for TFP. Additionally, innovative therapeutic approaches, such as mindfulness-based therapies, virtual reality therapy, and neurostimulation techniques, show promise in expanding the range of available treatments. To ensure the safety and efficacy of these novel approaches, well-designed randomized controlled trials and comparative effectiveness studies are warranted. Moreover, personalized interventions based on biomarkers and genetic factors can enhance treatment outcomes. Bridging the research-practice gap is essential in ensuring evidence-based interventions are accessible to those in need. By addressing these research gaps and challenges, the field of PTSD treatment can continue to progress, offering improved outcomes and tailored interventions that bring hope and healing to individuals living with PTSD.
